# 

**Published:** 1899-04-29

**Authors:** 


					THE HOSPITAL. ?The ^andard of highest purity."? Lancet, April 29th, 1899. fj
c- CADBURYS *+? U B* CADBURYS
coooa 1L M> M JIc?. cocoa
ABSOLUTELY PURE. ^ NO DRUGS OR ALKALIES USED.
No. 657>IVol. XXYI. [fgpaper!] Saturday,
April 29ih, 1899.
Subscription Terms,
eee p. 74,
] P&ICE/Tyft^^NCE.

				

